# A Technology Platform to Test the Efficacy of Purification of Alginate

**DOI:** 10.3390/ma7032087

**Published:** 2014-03-12

**Authors:** Genaro A. Paredes-Juarez, Bart J. de Haan, Marijke M. Faas, Paul de Vos

**Affiliations:** Department of Pathology and Medical Biology, Section of Immunoendocrinology, University Medical Center Groningen, University of Groningen, Hanzeplein 1, EA11, 9700 RB Groningen, The Netherlands; E-Mails: b.j.de.haan@umcg.nl (B.J.H.); m.m.faas@umcg.nl (M.M.F.); p.de.vos@umcg.nl (P.V.)

**Keywords:** alginate purification, pathogen-associated molecular patterns (PAMPs), microencapsulation, immunology

## Abstract

Alginates are widely used in tissue engineering technologies, e.g., in cell encapsulation, in drug delivery and various immobilization procedures. The success rates of these studies are highly variable due to different degrees of tissue response. A cause for this variation in success is, among other factors, its content of inflammatory components. There is an urgent need for a technology to test the inflammatory capacity of alginates. Recently, it has been shown that pathogen-associated molecular patterns (PAMPs) in alginate are potent immunostimulatories. In this article, we present the design and evaluation of a technology platform to assess (i) the immunostimulatory capacity of alginate or its contaminants, (ii) where in the purification process PAMPs are removed, and (iii) which Toll-like receptors (TLRs) and ligands are involved. A THP1 cell-line expressing pattern recognition receptors (PRRs) and the co-signaling molecules CD14 and MD2 was used to assess immune activation of alginates during the different steps of purification of alginate. To determine if this activation was mediated by TLRs, a THP1-defMyD88 cell-line was applied. This cell-line possesses a non-functional MyD88 coupling protein, necessary for activating NF-κB via TLRs. To identify the specific TLRs being activated by the PAMPs, we use different human embryonic kidney (HEK) cell-line that expresses only one specific TLR. Finally, specific enzyme-linked immunosorbent assays (ELISAs) were applied to identify the specific PAMP. By applying this three-step procedure, we can screen alginate in a manner, which is both labor and cost efficient. The efficacy of the platform was evaluated with an alginate that did not pass our quality control. We demonstrate that this alginate was immunostimulatory, even after purification due to reintroduction of the TLR5 activating flagellin. In addition, we tested two commercially available purified alginates. Our experiments show that these commercial alginates contained peptidoglycan, lipoteichoic acid, flagellin, and even lipopolysaccharides (LPS). The platform presented here can be used to evaluate the efficacy of purification procedures in removing PAMPs from alginates in a cost-efficient manner.

## 1. Introduction

Alginate is a commonly applied polymer in encapsulation research. Alginate was originally introduced for microencapsulation of pancreatic islets [1] but is now applied for immunoprotection of many other endocrine and recombinant cells. Currently it is applied for the delivery of therapeutic products [2,3], such as growth hormone and human clotting factor IX.9 production [4–7]. Also, it is used in bioartificial kidneys [8], for protection of hepatocytes [9], and for bioartificial parathyroids [10]. Alginate is being applied for both macro- and microencapsulation [11] as well as for enveloping cells in other fields of tissue engineering [12].

Alginate is a linear polysaccharide composed of 1,4′-linked β-d-mannuronic acid (M) and α-l-guluronic acid (G) residues in different sequences [13]. The ratio of G and M blocks is dependent on the source of algae used for alginate extraction [14]. Alginates with a high-G content are preferred for applications where a more rigid structure is required [15]. Alginates with a higher M content are preferred for applications where pliable structures are prioritized. This versatile property of alginate is caused by the higher affinity of the G residues for divalent ions [16]. The alginate that is most commonly applied for cell encapsulation is harvested from brown algae such as *Laminaria hyperborea*, *Macrocystis pyrifera*, *Laminaria digitata*, *Ascophyllum nodosum*, *Laminaria japonica*, *Eclonia maxima*, *Lessonia nigrescens*, *Durvillea antarctica* and *Sargassum* spp. [17].

A persistent issue in application of alginate is the different degree of biocompatibility, which seems to be laboratory dependent [18,19]. Different factors such as the use of different types of alginates [20], the type of coating [13] and variations in purity of alginate have been shown to be a major cause of the variations in success of the capsules in terms of biocompatibility and acceptance by the host [21–23]. Also, items such as capsule porosity is a criteria for cell survival [16,24] as well as stiffness that might influence cell differentiation [2]. Purification of alginate is reported to reduce inflammatory responses against alginate based capsules but many groups have difficulties in reliably producing ultrapure alginates [20,25,26]. Another issue is that many used procedures to purify alginate have been published [22,27] but techniques to predict whether the purification is efficacious are lacking.

Recently, we demonstrate that pathogen-associated molecular patterns (PAMPs) in alginates are one of the dominant molecules responsible for tissue responses after implantation of encapsulated tissues or cells [28]. PAMPs are small molecular motifs found on groups of microorganisms and can be recognized by Toll-like receptors (TLRs) and other pattern recognition receptors (PRRs) on cells of the innate immune system [29]. In the present study, we applied this knowledge to design a technology platform that can be used to evaluate the efficacy of purification procedures for alginate. The platform is composed of several fast and cost-effective procedures designed to (i) stepwise assessment of the immunostimulatory capacity of alginate, (ii) wherein the purification process the PAMPs are removed, and (iii) which TLRs and their ligands are being involved and might result in a smaller sample size required for biocompatibility and animal testing.

## 2. Results

### 2.1. Design of a Technology to Identify PAMPs in Alginate

The very first step in the platform was a fast screening for NF-κB based immunostimulation of THP1-XBlue™-MD2-CD14 monocytes by the alginate samples. If pathogen-associated molecular patterns (PAMPs) are absent in the alginate, the cells will not be activated, but when PAMPs in the range of picograms are present the cells will react [28,30].

The next step was to determine whether the PAMPs are ligands for Toll-like receptors (TLRs) NOD receptors or other pattern recognition receptors (PRRs). To this end, we tested the alginate on THP1-XBlue™-MD2-CD14–defMyD88 cells. These cells have a nonfunctional MyD88 signaling. All TLRs with the exception of TLR3 require MyD88 for NF-κB activation. If the cells give no signal—which up to now happened with all alginates tested—we continued to the next step.

**Figure 1.**
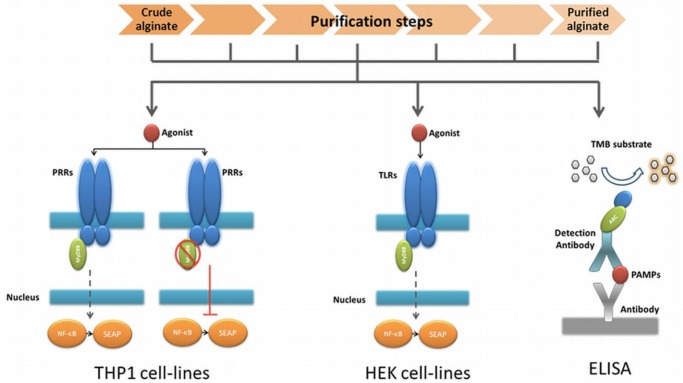
Technology to assess the immunostimulatory capacity of alginate, to determine where in the purification process the pathogen associated molecular patterns (PAMPs) are removed, and which Toll-like receptors (TLRs) and ligands are being involved. We applied a THP1 monocytic cell-line in the first step to determine the immunostimulatory capacity of the alginate. Next, we applied a THP1 with a non-functional MyD88 coupling protein. This allowed us to confirm that the immune stimulation is PRR dependent. In the next step, HEK cell-lines that possess the specific receptors were applied. This approach allows for scaling down the number of candidate PAMPs that might be responsible for the response. The last step was application of an enzyme-linked immunosorbent assays to identify the specific PAMPs.

In order to identify the exact type and amount of PAMP in a stepwise fashion in the alginate, we employed a relatively inexpensive test involving transgenic human embryonic kidney (HEK) cells with one specific TLR. Also, these cells carry a NF-κB reporter allowing fast identification of the TLR involved. The last step in the platform was application of ELISAs to identify the specific PAMP in the alginate (Figure 1).

The advantage of the above described work flow is that it avoids a time consuming and expensive screening for non-specific PAMPs by ELISAs only. Another advantage is that it provides data on immunostimulatory capacity of the alginate preparations.

### 2.2. Application of the Technology to Test the Efficacy of Alginate Purification Procedures

The most commonly applied procedure to purify alginate is chemical extraction of contaminant [25,31]. As outlined in the materials and methods, our published procedure was composed of six distinct steps that were originally introduced as extraction steps for differentially charged proteins [21]. The above-presented technology was applied to demonstrate the efficacy of the purification procedure in removing PAMPs. In order to demonstrate the efficacy of the technology for screening the adequacy of the purification procedure, we showed here an example of a purification-run in which the end product contained a novel introduced contamination that was not present in the crude alginate. This alginate had not passed the screening as a result of the screening and had not been applied for encapsulation research *in vivo*.

**Figure 2.**
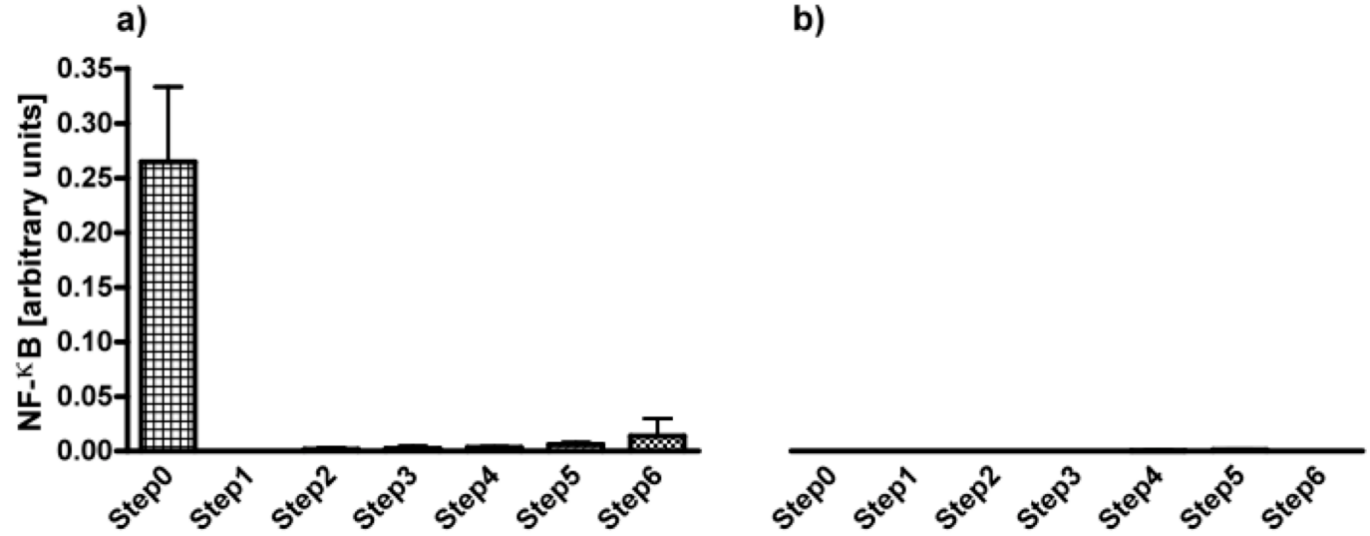
(**a**) Activation of NF-κB in THP1 reporter cell line to determine the immunostimulatory capacity of alginate in the six different steps of purification. (**b**) This activation was completely MyD88 dependent as it was not present in THP1 cell-line that has a non-functional MyD88. Activation of NF-κB was almost completely gone after the first steps of purification. Values are presented as mean ± SD (*n* = 5). All the purification steps presented a statistically significant response (*p* < 0.001) when compared with crude alginate (step 0) or with purified alginate (step 6). LPS (1 μg/mL) was used as positive control for the THP1-cell line, and induced an activation of NF-κB of 1.039 ± 0.081. For the THP1 cell line presenting a MyD88 non-functional protein, Tri-DAP (10 µg/mL) was used as a positive control, with a NF-κB activation of 0.053 ± 0.006.

Alginates from all six steps were applied for testing on THP1-XBlue™-MD2-CD14 monocytes. As shown in Figure 2a, the first step, *i.e.*, filtration was the most efficacious step in removing THP1 immunostimulating contaminations. The immunostimulatory capacity remained low after the next steps but gradually increased toward step 6.

Next, the samples were cultured with THP1-XBlue™-MD2-CD14–defMyD88 cells that lack a functional TLR signaling. As shown in Figure 2b, there was no activation of NF-κB in the absence of MyD88 illustrating that the activation is TLR dependent.

### 2.3. Stimulation of TLR2, 4, 5, and 9 by Impurities in Alginates during Purification Steps

To identify the specific TLRs involved, we used human embryonic kidney (HEK) cells expressing the specific MyD88 dependent Toll-like or Nod-like receptors. Table 1 shows the percentage change when HEK cell-lines with TLR2, 3, 4, 5, 7, 8, and 9, and NOD1 and 2 were stimulated with the (partly) purified alginates. Specific activation was found for TLR2, 4, 5 and 9 but not for TLR3, 7 and 8 and neither for NOD1 and 2. Activation was reduced to that of the negative controls after the filtration steps for TLR9, but not for TLR2, TLR4 and TLR5. TLR2 was activated from steps 1–5 (*p* < 0.05). It was deleted in the final purification step, *i.e.*, step 6. TLR4 was activated from step 0–6 (*p* < 0.01). TLR5 was activated by alginate obtained from step 0 and 2 (*p* < 0.01), but was no longer activated by alginate obtained as of step 3. A statistical significant activation of TLR9 was found during the last step of purification, illustrating that novel PAMP contaminations can be introduced during the purification procedures (*p* < 0.01).

### 2.4. PAMPs Flagellin and Peptidoglycan are Present during the Purification Steps

**Figure 3.**
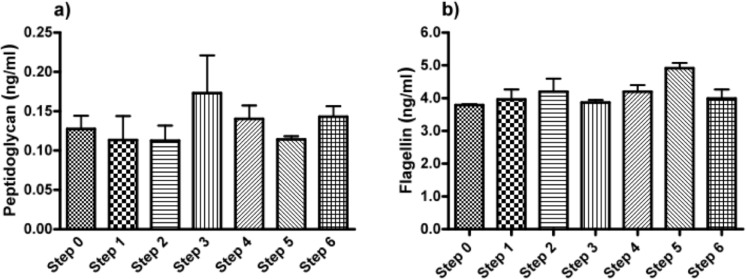
(**a**) Peptidoglycan and (**b**) flagellin concentration in the different purification steps of intermediate-G alginate. Lipoteicoic acid (LTA; TLR2 ligand), lipopolysaccharides (LPS; TLR4 ligand) and unmethylated CpG (CpG-ODN; TLR9 ligand) were not detected. Experiments were performed in triplicates.

As a last step, we applied ELISAs directed against the ligands of the TLRs found in the HEK cell-lines based assays. To this end, we screened for common and specific molecules known for triggering an immune response in the stimulated TLR2 (lipoteichoic acid, LTA; peptidoglycan, PG), TLR4 (lipopolysaccharides, LPS), TLR5 (Flagellin) and TLR9 (unmethylated CpG, CpG-ODN). In crude alginate and in all the steps of purification, we found PG and Flagellin, which were responsible for activation of HEK cell-lines carrying hTLR2 and 5. Figure 3 shows both PG and Flagellin were found as of the beginning and could not be totally removed by the purification steps. We could not find LTA, CpG-ODN or LPS (Figure 3).

### 2.5. Immunostimulatory Capacity of Commercially Available Purified Alginates

**Figure 4.**
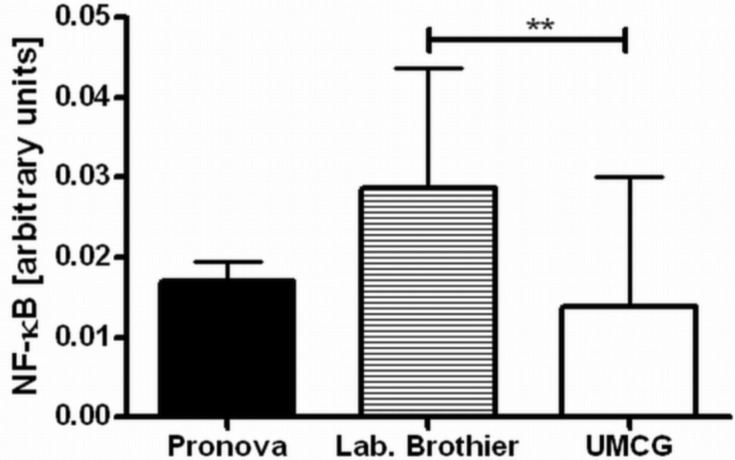
Immune activation by commercially available ultrapure alginate and alginate as shown in the previous graphs. The right bar indicated our own procedure of purification, *i.e.*, how it is performed at the University Medical Center Groningen (UMCG).Values are presented as mean ± SD. LPS was used as a positive control, which induced a NF-κB activation of 2.070 ± 0.135. The difference between our purification proceeding and the one from Laboratoires Brothier was statistically significant (*p* < 0.01, **).

Next, we applied our toolbox to test the adequacy of two commercially available alginates that are marketed as purified alginate. These were alginates purchased from Pronova (Pronova UltraPure medium viscosity (MVG), G/M ratio ≥ 1.5, G content ≥ 60%, approximate *M*_w_ > 200 kDa, endotoxins ≤ 100 EU/g, viscosity > 200 mPa·s) and Les Laboratoires Brothier (now Kimica Algin High G I-3G, viscosity 300–400 mPa·s). As shown in Figure 4, both alginates still had immunostimulatory capacity. When compared to the alginate mentioned in Figure 2a, the commercial alginates were equally or even more immunostimulatory (Figure 4).

**Figure 5.**
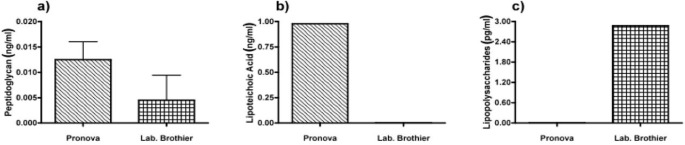
Presence of (**a**) peptidoglycan (PG; TLR2 ligand); (**b**) lipoteichoic acid (LTA; TLR2 ligand); (**c**) lipopolysaccharides (LPS, TLR4 ligand) in commercially available ultrapure alginate of Pronova and Laboratoires Brothier.

Due to immune activation found in commercially available alginate, we applied ELISAs to identify the type of contamination responsible for immunostimulation. Alginate from Pronova contained peptidoglycan and lipoteichoic acid (TLR2 ligands), while the alginate from Les Laboratoires Brothier contained peptidoglycan (TLR2 ligand) and lipopolysaccharides (TLR4 ligand) (Figure 5). This demonstrated the need of a platform as presented here to test also commercially obtained alginates.

## 3. Discussion

In this study, we present a novel technology platform to test for the presence of (i) immunostimulatory capacity of alginate, (ii) where in the purification process PAMPs are removed, and (iii) which TLR and ligands are involved. The principal applicability of the platform was evaluated with an alginate that normally would not pass our criteria for application in *in vivo* studies to demonstrate its potential and usefulness in testing efficacy of our procedure. Prior to this research, only implantation studies involving rats and mice have been used. This is time-consuming, presents ethical issues, and is costly. Our approach is less labor intensive, relatively fast, and affordable.

Also, our platform allows for fast screening of commercially available alginates which are presently being applied and are considered to be ultrapure. The samples that we bought were actually not ultrapure and already gave immunostimulatory signals as of the first step. The Pronova alginate is the most commonly applied alginate [14,24,27,32–34]. In our test sample, it was found to contain peptidoglycan (PG) and lipoteichoic acid (LTA). According to the manufacturer, it is ultrapure and below reliable quantification of endotoxin levels by the Limulus amebocyte lysate (LAL) assay. Here, it is shown that the level of contaminant is enough to induce an immune reaction and detectable with our platform. The other alginate from Les Laboratoires Brothier contains PG and lipopolysaccharides (LPS). With our standards, it would not pass the criteria for *in vivo* application. Alginates should be free of these contaminants when applied *in vivo* [20,22,27].

Prior to our research, the LAL assay was applied to test for presence of endotoxins [35]. LAL is an extract of blood cells from the horseshoe crab, *Limulus polyphemus*. We recommend use of more specific measures such as the ELISA approach instead of LAL for the following reason: LAL has been considered to react mainly with bacterial lipopolysaccharide (LPS). Although it has recently been shown that also the presence of lipoteichoic acid (LTA) and peptidoglycan (PG) [35] may result in positive LAL assays, it is unknown how sensitive the assay for other endotoxins or PAMPs is. In addition, our platform provides insight concerning which PAMPs are still present in alginate, which gives researchers the opportunity to design specific means to remove the molecules.

As shown in the present study, stepwise filtration towards 0.45 μm filters is an effective method to remove most of the PAMPs present in crude alginate. At the same time, we demonstrate with the example that new contaminations with PAMPs can be introduced during the purification course. The causes of these new contaminations with PAMPs may be multifactorial. The applications of forceps that might contain bacteria, funnels that have been previously flushed with demi-water or exposure to non-sterile atmospheric air are sources for bacterial contaminations and thus PAMPs. It is crucial to use endotoxin free water for all solutions and when washing all equipment in order to avoid introduction of contaminants to reproduce a high purity alginate.

A common issue in the application of our platform is that specific TLRs are being activated while the ligand cannot be detected with ELISA. This should be explained as follows: Many TLRs have more ligands [30] than the classically described such as LTA for TLR2, CpG-ODN for TLR9 or LPS for TLR4. We have to be aware that activation can occur via other molecules not described yet [36–38]. It might even be specific alginate molecules such as small molecular poly-MM that can bind to TLR4 and CD14 [39]. We believe, however, that these molecules should be removed in order to facilitate biocompatibility. Therefore, it is important to not only apply ELISAs for screening purity, but also test whether TLRs are activated by the purified alginate.

Most purification procedures for alginate were originally designed to remove proteins [22]. Our data indicates that proteins are not very immunostimulatory as evidenced by the samples from step one where no protein extraction was performed and there was barely an immunostimulatory on THP1 cells. This supports our previous findings [28] but it does not seem to corroborate the findings of Ménard *et al.* [27] who study the protein content of both commercial and own purified alginates and found a correlation with immunostimulatory capacity of the studied alginates. It is, however, difficult if not impossible to compare our results with Ménard *et al.* [27] since knowledge concerning the role of PAMPs was not available at the time their research was conducted. In our research, when PAMPs were absent, the proteins in the alginate are not immunostimulatory.

The lack of immunostimulatory effects of alginates still containing proteins does not suggest that there is no need to remove proteins. As shown in previous studies [40], the formation of an adequate, persisting membrane of poly-l-lysine (PLL) is a delicate matter. Proteins present in crude alginate make the process of binding of PLL more complex and less predictable and should therefore be avoided.

In recent reviews [41,42], it has been emphasized that the area of tissue engineering in which alginate is commonly applied is characterized by a low degree of reproducibility and lack of assays to compare materials between laboratories. This has been one of the principle reasons for designing and publishing this research. It is our hope that the current research will contribute to a better understanding of the composition and immunostimulatory capacity of the alginates applied in the different studies that will make interpretation and comparison more adequate.

## 4. Materials and Methods

### 4.1. Chemicals

Intermediate-G alginate (ISP Alginates Ltd., Ayrshire, UK) has been used (42% G-chains, 58% M-chains, 23% GG-chains, 19% GM-chains, 38% MM-chains, *M*_n_ = 428 kDa) for studies on efficacy of purification. The composition of these alginate samples was studied by proton nuclear magnetic resonance (^1^H-NMR). Commercially available purified alginates from Les Laboratoires Brothier (Paris, France) and Pronova™ (FMC BioPolymer, Sandvika, Norway) were applied to compare the degree of purity and content of pathogen-associated molecular patterns.

QUANTI-Blue™ (InvivoGen, Toulouse, France) is a medium with a colorimetric enzyme used to detect activity of any alkaline phosphatase. QUANTI-Blue^™^ medium turns purple-blue in the presence of alkaline phosphatase (SEAP) and can be quantified using a spectrophotometer at 620–655 nm.

### 4.2. Purification

A commonly applied chemical-purification method is applied [21]. This chemical extraction method does not influence the alginate gelling condition [22]. The purification procedure was categorized into six distinct steps. After each step a sample was taken to assess the immunostimulatory capacity in order to gain insight in the efficacy of removing immunostimulatory contaminants in the alginate.

Step 0: a sample from crude non-purified alginate was taken.

Step 1: crude sodium alginate was dissolved at 4 °C in a 1 mM sodium ethylene glycol tetraacetic acid (EGTA) solution to a 1% solution under constant stirring. Subsequently, the solutions were filtered over successively 5.0, 1.2, 0.8, and 0.45 μm filters (Whatman^®^, Dassel, Germany). During this filtration step, all visible aggregates were removed.

Step 2: the pH of the solution was lowered to 3.5 by addition of 2 N HCl + 20 mM NaCl. The solution was kept on ice to prevent hydrolysis of alginate. The next step was to slowly lower the pH from 3.5 to 2.0. This is associated with gradual precipitation of alginate as alginic acid [43]. Routinely, the solutions were brought at a pH of 2.0 and subsequently filtered over a Buchner funnel (pore size 1.5 mm) to wash out non-precipitated contaminants. To extend the washout of non-precipitated contaminants, the precipitate was brought in 0.01 N HCl + 20 mM NaCl, vigorously shaken, and filtered again over the Buchner funnel. This washing procedure was performed three times and another sample was taken.

Step 3: proteins were removed by extraction with chloroform/butanol [44]. The alginic acid was suspended in 100 mL of 0.01 N HCl + 20 mM NaCl and supplemented with chloroform (20 mL at each 100 mL alginate solution) and 1-butanol (5 mL at each 100 mL alginate solution). The mixture was vigorously shaken for 30 min and filtered over the Buchner funnel. This chloroform/butanol extraction was performed three times and a third sample was obtained after the last extraction.

Step 4: the alginic acid was brought in water and slowly dissolved by gradually raising the pH to 7.0 by slow addition of 0.5 N NaOH + 20 mM NaCl over a period of at least one hour. The alginate solution obtained was subjected to a chloroform/butanol extraction to remove those proteins which can only be dissolved in chloroform/butanol at neutral pH [44]. The solution was vigorously shaken in a mixture of chloroform (20 mL at each 100 mL alginate solution) and 1-butanol (5 mL at each 100 mL alginate solution) for 30 min. The mixture was centrifuged for 5 min at 1800 rpm, which induced the formation of a separate chloroform/butanol phase, which was removed by aspiration. The extraction was repeated once and then a sample was taken.

Step 5: the last step is precipitation of the alginate with ethanol [43]. To each 100 mL of alginate solution we added 200 mL of absolute ethanol. After an incubation period of 10 min, all alginate had precipitated. The alginate was filtered over the Buchner funnel and washed two times with absolute ethanol and a sample was obtained.

**Figure 6.**
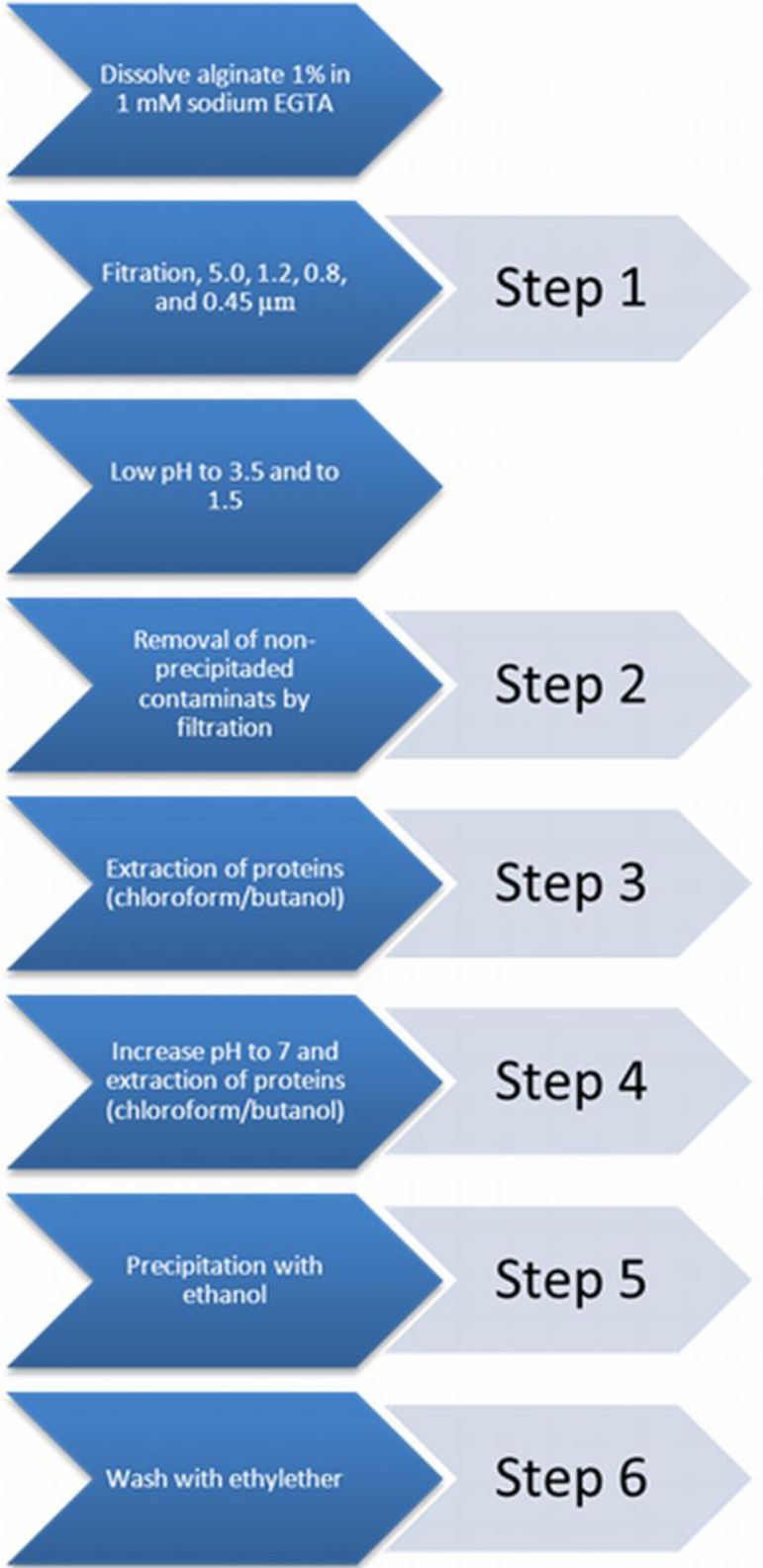
The applied chemical extraction of contaminants of alginate and the six distinct steps in the purification procedure.

Step 6: subsequently, the alginate was washed three times with ethylether and the last sample of purified alginate was taken (Figure 6).

All the samples of alginate were freeze-dried (Freezone 2.5 Plus, Labconco, Kansas, MO, USA) overnight for immunostimulation and enzyme-linked immunosorbent assays (ELISAs).

### 4.3. Immunostimulation by Alginates and Identification of Pattern Recognition Receptors Involved

To determine the immunostimulatory capacity of samples of alginates obtained from the different steps of purification, we dissolved the alginate at a concentration of 0.3% (w/v) in a solution of Krebs-Ringer-Hepes (KRH) with an appropriate osmolarity. Subsequently, alginate solutions were co-incubated with different cell lines (InvivoGen) expressing pattern recognition receptors (PRRs) under the control of a reporter gene. First, the sample was co incubated with THP1-XBlue™-MD2-CD14. THP1-XBlue™-MD2-CD14 expresses all Toll-like receptors (TLRs) [13,28,30]. We applied a NF-κB/AP-1 transcription that endogenously expresses all TLRs and additional inserts for the co-signaling molecules CD14 and MD2. This facilitates TLR-mediated responses [45]. In addition, we applied a THP1 cell expressing only a truncated, non-functional form of the TLR adapter MyD88 (THP1-XBlue™-defMyD). MyD88 is an essential coupling messenger in the cascade from TLR and nucleotide-binding oligomerization domain receptors (NODs) activation towards NF-κB activation. Also, we applied human embryonic kidney (HEK)-cells overexpressing a specific TLR or NOD. The HEK-Blue™ cell-lines are produced by co-transfection of human TLR (2, 3, 4, 5, 7, 8 or 9), MD-2 and CD14 co-receptor genes into the HEK 293 cells. These are designed to identify the activation of specific human TLR2, 3, 4, 5, 7, 8 or 9 and NOD1 or 2 combined with AP-1 and a NF-κB reporter construct.

The cell-lines THP1-XBlue™-MD2-CD14 and THP1-XBlue™-defMyD88 cells were cultured in Roswell Park Memorial Institute (RPMI) 1640 culture medium (supplemented with 2 mM·l-glutamine, 1.5 g/L sodium bicarbonate, 4.5 g/L, 10 mM 4-(2-hydroxyethyl)-1-piperazineethanesulfonic acid (HEPES) and 1.0 mM sodium pyruvate, with 10% fetal bovine serum (deactivated phosphatases)). The medium also contains 100 μg/mL Normocin™ and Pen-Strep (50 U/mL–50 μg/mL). The cells were plated at a concentration of 1 × 10^6^ cells/mL in 96-wells plates. Cells were stimulated with samples of alginates obtained from the different steps of purification (*n* = 5) and cultured overnight at 37 °C and 5% CO_2_. As positive controls, lipopolysaccharide from *Escherichia coli* K12 strain (LPS-EK Ultrapure 10 μg/mL, InvivoGen) was used for the THP1-XBlue™-MD2-CD14 cell line and l-Ala-γ-D-Glu-mDAP (Tri-DAP 10 μg/mL, InvivoGen) for the THP1-XBlue™–defMyD88 cell line. RPMI 1640 culture medium served as was used as a negative control. SEAP was quantified by using QUANTI-Blue™ (InvivoGen).

HEK-Blue™ cells were suspended in Dulbecco’s Modified Eagle’s culture medium (DMEM) supplemented with 4.5 g/L glucose, 10% (v/v) fetal bovine serum (deactivated phosphatases), Pen-Strep (50 U/mL–50 μg/mL), 100 μg/mL Normocin™ and 2 mM l-glutamine). Cells were seeded at the following concentrations; at 280.000 cells/mL (hTLRs 2 and NOD 1), 140.000 cells/mL (hTLRs 4, 5 and NOD 2), 220.000 cells/mL (hTLRs 7 and 8), and 450.000 cells/mL (hTLR 9), in 96-wells plates according to standard protocols [28,32]. Each well was stimulated with samples of alginates in different steps of purification (*n* = 5) and cultured overnight at 37 °C and 5% CO_2_. DMEM culture medium served as negative control. TLRs’ signaling was always confirmed using the appropriate TLR or NOD ligand. These were TLR2, synthetic diacylated lipoprotein (FSL-1); TLR3, polyinosine-polycytidylic acid (poly(I:C) high molecular weight (HMW)); TLR4, ultrapure lipopolysaccharide from *E. coli* K12 strain (LPS-EK Ultrapure); TLR5, recombinant flagellin from *Salmonella typhimurium* (RecFLA-ST); TLR7, 9-benzyl-8 hydroxyadenine (CL264); TLR8, 20-mer phosphorothioate protected single-stranded RNA oligonucleotide (ssRNA 40); TLR9, unmethylated CpG dinucleotides (Class B CpG oligodeoxynucleotide (ODN 2006); NOD1, l-Ala-γ-d-Glu-mDAP (Tri-DAP); and NOD2, synthetic derivative of muramyl dipeptide (L18-MDP). NF-κB activation was quantified by the SEAP activity using QUANTI-Blue™ (Invivogen). Experiments were repeated at least five times.

### 4.4. Measurement of Specific Pathogen-Associated Molecular Patterns (PAMPs)

The presence of lipoteichoic acid (LTA; TLR2 ligand) was measured using a Human Lipoteichoic Acid ELISA Kit (Wuxi Donglin Sci&Tech Development Co. Ltd., Wuxi, Jiangsu, China). This ELISA is based on the competitive binding enzyme immunoassay technique. The samples were analyzed in triplicate, according to assay protocol. The enzyme-substrate reaction is terminated by the addition of 0.05 mL of sulfuric acid solution and measured spectrophotometrically at a wavelength of 450 nm. All the incubations were performed at 37 °C and 5% CO_2_.

The content of lipopolysaccharides (LPS; TLR 4 ligand) was measured using a Human Lipopolysaccharides ELISA Kit (Cusabio, Wuhan, China). For this assay, the quantitative sandwich enzyme immunoassay technique was employed, according to assay protocol. The optical density was measured at 450 nm within 5 min after the reaction was stopped. All the incubations were performed at 37 °C and 5% CO_2_.

Quantification of flagellin (TLR5 ligand), human peptidoglycan (PG, TLR2 ligand), and unmethylated dsDNA (CpG-ODN, TLR9 ligand) was done with a Human Flagellin ELISA Kit, a Human Peptidoglycan (PG) ELISA kit, and a Human CpG oligodeoxynucleotide (CpG-ODN) ELISA kit (Qayee-Bio, Shanghai, China) according to assay protocols. The plates were read in a spectrophotometer at a relative optical density of 450 nm within 15 min after adding the stop solution. All the incubations were performed at 37 °C and 5% CO_2_.

### 4.5. Statistical Analysis

Results are expressed as mean ±SD. Statistical comparisons were made with the Mann Whitney U test using the GraphPad Prism 5.00 software (GraphPad Software, Inc., La Jolla, CA, USA). A *p* value<0.05 was considered statistically significant.

## 5. Conclusions

In the present study, we evaluated a technology platform designed for alginate to assess (i) the immunostimulatory capacity of alginate or its contaminants, (ii) wherein the purification process the PAMPS are removed or reintroduced, and (iii) which TLR and ligands are involved. We demonstrate the applicability by applying a purification run designed by our group and by testing two commercially available alginates which were marketed as ultrapure. These commercial ultrapure alginates contained ligands like peptidoglycan, lipoteichoic acid, flagellin, and even LPS. The activation of the immune system via TLRs and release of NF-κB was demonstrated in our platform.

## Figures and Tables

**Table 1.** Percent change of NF-κB in HEK cell-lines with specific Toll-like receptors (TLRs) when stimulated with alginate in the different steps of purification. Contaminations can activate hTLR2, 4, 5 and 9. Values are presented as percentages. Each cell-line was stimulated with an appropriate positive control with different values of NF-κB activation: (a) TLR2 (FSL-1, 1 μg/mL, 1.272 ± 0.057); (b) TLR3 (Poly(I:C) HMW, 5 μg/mL, 0.217 ± 0.015); (c) TLR4 (LPS-EK Ultrapure, 100 ng/mL, 0.270 ± 0.009); (d) TLR5 (RecFLA-ST, 100 ng/mL, 2.626 ± 0.023); (e) TLR7 (Imiquimod (R837), 50 μg/mL, 0.154 ± 0.056); (f) TLR8 (ssRNA 40, 50 μg/mL, 0.065 ± 0.009); (g) TLR9 (ODN 2006, 100 μg/mL, 1.088 ± 0.051); (h) NOD1 (Tri-DAP, 10 μg/mL, 0.458 ± 0.068); (i) NOD2 (L18-MDP, 100 ng/mL, 0.504 ± 0.069). DMEM 1640 growth media was used in all the cell lines as a negative control. The threshold for biologically relevant was higher than 0.01 (arbitrary units). This level is the minimum value that purified alginate in solution can reach. Values are presented as percent change; *p* < 0.05 (*) and *p* < 0.01 (**).

HEK cell-line	Purification steps							Positive control
	0	1	2	3	4	5	6	
TLR2	−20.2	143.7 ^*^	149.6 ^*^	108.4 ^*^	115.1 ^*^	155.1 ^*^	−30.8	1461.7
TLR3	0.5	−12.2	2.9	2.6	4.4	6.2	4.9	418.8
TLR4	108.1 ^**^	70.5 ^**^	43.4 ^**^	38.0 ^**^	43.0 ^**^	65.2 ^**^	67.7 ^**^	324.1
TLR5	20.1 ^**^	−3.8	28.7 ^**^	1.8	−8.3	12.0	−4.5	1878.9
TLR7	4.86	11.15	−5.42	−4.83	0.58	1.90	−0.85	104.10
TLR8	−31.64	−1.83	6.00	−6.74	−1.22	4.74	−28.35	11.11
TLR9	6.35	2.40	−1.52	−2.46	−2.13	5.83	103.5 ^**^	1033.33
NOD1	7.18	−5.42	−1.00	−0.72	1.28	2.53	4.13	818.94
NOD2	−4.44	−12.00	−13.47	−11.28	−14.45	−10.87	−7.46	451.05
